# Hydroxyurea Optimization through Precision Study (HOPS): study protocol for a randomized, multicenter trial in children with sickle cell anemia

**DOI:** 10.1186/s13063-020-04912-z

**Published:** 2020-11-27

**Authors:** Emily R. Meier, Susan E. Creary, Matthew M. Heeney, Min Dong, Abena O. Appiah-Kubi, Stephen C. Nelson, Omar Niss, Connie Piccone, Maa-Ohui Quarmyne, Charles T. Quinn, Kay L. Saving, John P. Scott, Ravi Talati, Teresa S. Latham, Amanda Pfeiffer, Lisa M. Shook, Alexander A. Vinks, Adam Lane, Patrick T. McGann

**Affiliations:** 1 Indiana Hemophilia and Thrombosis Center, Indianapolis, IN USA; 2 Division of Hematology/Oncology/BMT, Nationwide Children’s Hospital, Columbus, OH USA; 3 Center for Innovation in Pediatric Practice, Nationwide Children’s Hospital Research Institute, The Ohio State University, Columbus, OH USA; 4 Division of Hematology/Oncology, Boston Children’s Hospital, Boston, MA USA; 5 Harvard Medical School, Boston, MA USA; 6 Division of Clinical Pharmacology, Cincinnati Children’s Hospital Medical Center, Cincinnati, OH USA; 7 University of Cincinnati College of Medicine, Cincinnati, OH USA; 8 Pediatric Hematology, Children’s Hospitals and Clinics of Minnesota, Minneapolis and St. Paul, MN USA; 9 Division of Hematology, Cincinnati Children’s Hospital Medical Center, 3333 Burnet Ave, MLC 7015, Cincinnati, OH 45229 USA; 10 Department of Pediatrics, Division of Pediatric Hematology, Oncology, and Stem Cell Transplantation, Cohen Children’s Medical Center of New York, New Hyde Park, NY USA; 11 Case Western Reserve University School of Medicine, Department of Hematology and Oncology, University Hospitals Rainbow Babies and Children’s, Cleveland, OH USA; 12 Emory University School of Medicine & Sickle Cell Disease Program, Children’s Healthcare of Atlanta, Atlanta, GA USA; 13 University of Illinois Peioria, Peoria, IL USA; 14 Medical College of Wisconsin, Milwaukee, WI USA; 15 Cleveland Clinic, Cleveland, OH USA

**Keywords:** Sickle cell anemia, Pharmacokinetics, Hydroxyurea, Pediatrics

## Abstract

**Background:**

Sickle cell disease (SCD) is a severe and devastating hematological disorder that affects over 100,000 persons in the USA and millions worldwide. Hydroxyurea is the primary disease-modifying therapy for the SCD, with proven benefits to reduce both short-term and long-term complications. Despite the well-described inter-patient variability in pharmacokinetics (PK), pharmacodynamics, and optimal dose, hydroxyurea is traditionally initiated at a weight-based dose with a subsequent conservative dose escalation strategy to avoid myelosuppression. Because the dose escalation process is time consuming and requires frequent laboratory checks, many providers default to a fixed dose, resulting in inadequate hydroxyurea exposure and suboptimal benefits for many patients. Results from a single-center trial of individualized, PK-guided dosing of hydroxyurea for children with SCD suggest that individualized dosing achieves the optimal dose more rapidly and provides superior clinical and laboratory benefits than traditional dosing strategies. However, it is not clear whether these results were due to individualized dosing, the young age that hydroxyurea treatment was initiated in the study, or both. The Hydroxyurea Optimization through Precision Study (HOPS) aims to validate the feasibility and benefits of this PK-guided dosing approach in a multi-center trial.

**Methods:**

HOPS is a randomized, multicenter trial comparing standard vs. PK-guided dosing for children with SCD as they initiate hydroxyurea therapy. Participants (ages 6 months through 21 years), recruited from 11 pediatric sickle cell centers across the USA, are randomized to receive hydroxyurea either using a starting dose of 20 mg/kg/day (Standard Arm) or a PK-guided dose (Alternative Arm). PK data will be collected using a novel sparse microsampling approach requiring only 10 μL of blood collected at 3 time-points over 3 h. A protocol-guided strategy more aggressive protocols is then used to guide dose escalations and reductions in both arms following initiation of hydroxyurea. The primary endpoint is the mean %HbF after 6 months of hydroxyurea.

**Discussion:**

HOPS will answer important questions about the clinical feasibility, benefits, and safety of PK-guided dosing of hydroxyurea for children with SCD with potential to change the treatment paradigm from a standard weight-based approach to one that safely and effectively optimize the laboratory and clinical response.

**Trial registration:**

ClinicalTrials.gov NCT03789591. Registered on 28 December 2018.

## Background

Sickle cell disease (SCD) is a devastating, inherited disorder of hemoglobin, affecting over 100,000 persons in the USA and millions worldwide [1–3]. The most severe forms of SCD, primarily HbSS and HbS/β^0^-thalassemia, account for a majority of the global cases of SCD and are collectively referred to as sickle cell anemia (SCA). Without early diagnosis and appropriate disease-modifying treatment, SCA results in significant morbidity and early mortality. The life-threatening clinical complications of SCA, including acute splenic sequestration crisis and stroke, frequently occur within the first decade of life [4, 5]. Organ damage caused by recurrent vaso-occlusion and tissue ischemia, which is often clinically silent, begins as early as 4–6 months of age, when fetal hemoglobin (HbF) begins to decline and sickle hemoglobin (HbS) starts to predominate [6–8]. Hydroxyurea has emerged as the primary disease-modifying therapy for SCA [9] with decades of evidence demonstrating the salutary laboratory and clinical effects, including reduction in both morbidity [10–12] and mortality [13–16]. The benefits of hydroxyurea are primarily due to its ability to increase the production of HbF [9, 17]. In response to a growing body of evidence demonstrating the benefits and safety of hydroxyurea, highlighted by the randomized, double-blind, placebo-controlled phase III BABY HUG trial, the National Heart, Lung, and Blood Institute (NHLBI) published guidelines in 2014 recommended that hydroxyurea be offered to all infants with SCA starting at 9 months of age, regardless of clinical severity [18]. For pediatric sickle cell centers that have successfully initiated hydroxyurea in infants and young children, there has been a notable improvement in the health of these children [19]. While the 2014 guidelines increased the early initiation of hydroxyurea, use remains concerningly low with recent data suggesting that less than 50% of children with SCA are prescribed this life-saving medication [20, 21].

In addition to the importance of early initiation of hydroxyurea to prevent SCA complications, our experience documents that hydroxyurea dosing is also a critically important determinant to optimize the clinical and laboratory effects of the medication. The clinical benefits of hydroxyurea are maximized when the HbF production is optimized and HbF effect is largely determined by degree of hydroxyurea exposure [22]. Even with modest HbF induction at lower doses, most patients have some clinical or laboratory benefits. However, optimal dosing is highly variable from patient to patient due to significant inter-patient variability in drug pharmacokinetics (PK) and pharmacodynamics (PD) [23–26]. Optimal doses range from 15 to 35 mg/kg/day, resulting in many patients receiving less than 50% their personalized, ideal dose. Recognizing this variability and using a precision medicine approach, we developed a model to individualize hydroxyurea dosing and optimize hydroxyurea response with the goal of minimizing the short- and long-term complications of SCA in young patients. With traditional hydroxyurea dosing, HbF levels ≥ 15–20% are considered a therapeutic success as they are often associated with a reduction in (but not elimination of) many clinical complications of SCA. A model-based publication suggests that HbF levels greater than 30% can achieve a “pharmacologic cure of most disease manifestations” [27, 28]. In the Therapeutic Response Evaluation and Adherence Trial (TREAT, ClinicalTrials.gov NCT02286154), we demonstrated that an individualized, PK-guided dosing strategy resulted in a more robust HbF response than seen with traditional weight-based dosing, as enrolled children achieved 30–50% HbF levels and had an absence of clinical SCA symptoms when initiating hydroxyurea at a PK-guided starting dose [29]. These unprecedented results in a population including children and young adults (6 months–21 years of age) have led to the hypothesis that early initiation and optimized hydroxyurea dosing actually can prevent, rather than only ameliorate, most short- and long-term sickle cell complications, including health-related quality of life.

Although encouraging, TREAT was a single-arm study at a single institution and many questions still remain. The TREAT cohort was very young in age with most children starting in the first 1–2 years of life while HbF levels remain high, it is not clear whether the robust HbF response observed was due to the early initiation of hydroxyurea (before genes involved in HbF expression may be fully silenced), whether this was a result of PK-guided dosing, aggressive dose escalation strategies, or perhaps a combination of the three. In addition, the ability to measure hydroxyurea concentrations is not widely available and the hydroxyurea PK model and PK-guided dosing strategy has not been used outside of Cincinnati Children’s Hospital Medical Center (CCHMC). Finally, the feasibility of performing PK studies in this population in the clinical setting including laboratory studies that require shipment of temperature-sensitive samples, and timely determination and implementation of a PK-guided hydroxyurea dosing regimen are important questions to answer to successfully implement personalized medicine approaches, such as this, among children with SCA in clinical practice. The Hydroxyurea Optimization through Precision Study (HOPS) is designed to address these specific key operational components. The multicenter trial includes several novel and innovative features, including individualized hydroxyurea dosing, sparse PK sampling requiring very small volumes of blood, a novel method of measuring hydroxyurea concentrations, and centralized initial dose selection with in a prospective, multicenter randomized trial.

## Methods/design

### Trial design

**Fig. 1:**
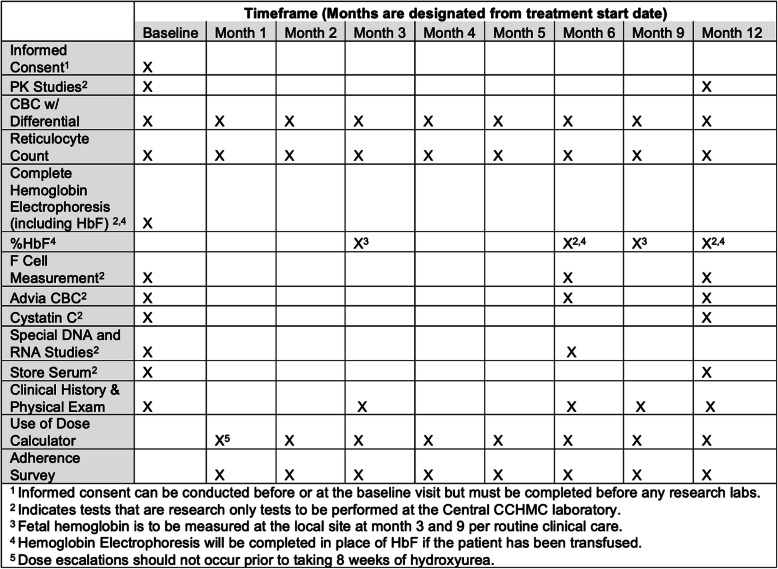
HOPS schedule of evaluations. All study-related procedures are outlined in this figure. The primary endpoint will be assessed at month 6 and the study will continue for a total of 12 months of hydroxyurea therapy

HOPS is a prospective, multicenter, randomized trial that aims to evaluate whether initial dosing of hydroxyurea using a novel PK-guided dosing strategy for children with SCA results in higher %HbF at 6 months compared to standard weight-based initial dosing with step-wise dose escalation. The multicenter design also allows for the validation of PK sample collection in young children with centralized PK analysis and dose selection. Participants are randomized in a 1:1 ratio to initially receive either a 20 mg/kg weight-based (Standard Arm) or a PK-guided (Alternative Arm) starting dose of hydroxyurea. Following initiation, a study-designed hydroxyurea dosing protocol will be used to escalate or reduce the initial hydroxyurea dose based on the laboratory data that will be collected through the time of the primary endpoint at month 6; the total study period is 12 months. Figure 1 outlines the schedule of events for participants in the study.

### Objectives

The primary objective of the study is to evaluate whether a PK-guided starting hydroxyurea dose results in a higher %HbF compared to standard weight-based (20 mg/kg) initial dosing for children with SCA. Secondary aims include careful investigation of the clinical, laboratory, and molecular determinants of the maximal hydroxyurea-induced HbF responses, as well as studies investigating changes in gene expression and regulation related to hydroxyurea starting dose and age.

### Study settings

**Fig. 2:**
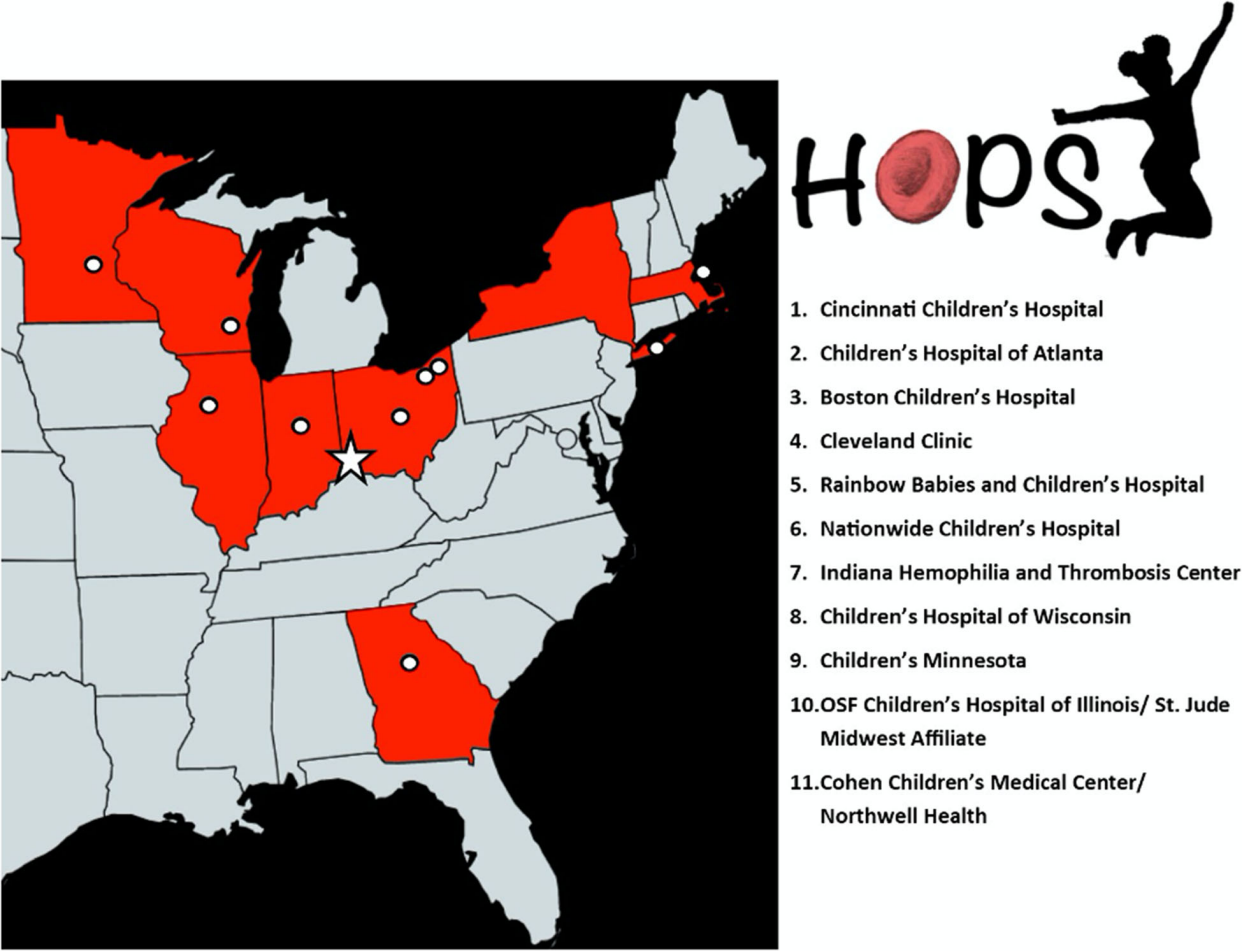
HOPS study sites. HOPS is a multi-center trial that is performed at 11 pediatric sickle cell centers across the USA. Cincinnati Children’s Hospital Medical Center serves as the study sponsor and Medical and Data Coordinating Center

Study participants are recruited from 11 pediatric sickle cell centers across the USA (Fig. 2). Most study sites were selected due to their involvement in the Sickle Treatment and Outcomes Research in the Midwest (STORM) regional network, led by CCHMC and established to improve care and outcomes for individuals with SCD living in Indiana, Illinois, Michigan, Minnesota, North Dakota, Ohio, South Dakota, and Wisconsin [30]. Additional study sites were included to ensure adequate enrollment and were selected based on previous collaborative relationships and investigator interest. Prior to the initiation of the study, there was significant variability in the number of patients (30 to greater than 500) and the proportion of children prescribed hydroxyurea (25–90%) at each site, mimicking the distribution of these patients across the USA and the known variability in hydroxyurea utilization. Prior to formal site selection, potential study sites completed a feasibility survey to assess patient volume, current hydroxyurea use, research capacity, and anticipated study enrollment. Subsequently, an in-person or virtual site training/initiation visit was performed, including review of study rationale and procedures as well as a comprehensive overview on the use of hydroxyurea therapy and sharing of the results from the TREAT trial. These site visits were well-received and allowed each study team to understand the rationale and strategy for dosing in the HOPS trial, which was different than the previous dosing strategies used for patients with SCA at these centers. Potential barriers to the recruitment of study participants or to the performance of study-related procedures were also reviewed to optimize the chance of smooth study success at each site.

### Eligibility criteria

Children with confirmed SCA (HbSS, HbSD, Hbβ^0^-thalassemia or other similarly severe phenotypes) who are initiating hydroxyurea therapy, following a discussion with their local clinical team, are eligible for study consideration. As each of these SCA genotypes is considered to have a similar phenotype and because we expect most enrolled participants to have HbSS disease, there will be no attempts to balance distribution of genotypes across the two study arms.

#### Inclusion criteria

1. Diagnosis of SCA (HbSS, HbSD, HbS/β^0^-thalassemia, or similarly severe SCA genotype)
2. Age 6 months to 21 years at the time of enrollment
3. Clinical decision by patient, family, and healthcare providers to initiate hydroxyurea therapy

#### Exclusion criteria

1. Current treatment with chronic, monthly blood transfusions or erythrocytapheresis. Of note, there are no restrictions regarding enrollment as to recent single blood transfusions. HbF is calculated as HbF/(HbF + HbS) to account for the presence of HbA. Children who are transitioning from chronic transfusions to hydroxyurea therapy are not eligible due to the difficulty in evaluating response with the overlap period of simultaneous transfusion therapy and hydroxyurea.
2. Treatment with hydroxyurea within the past 3 months
3. Hemoglobin SC disease, HbS/β^+^-thalassemia
4. Current treatment with other investigational sickle cell medications

### Recruitment and enrollment procedures

The study recruits patients who have decided to initiate hydroxyurea therapy based upon clinical indications and shared decision-making between the providers and the family. Informed consent is obtained by investigators or local research personnel at each study site. For children under 18 years of age, the parent or legal guardian provides written informed consent to join the study at the time of enrollment and older children sign assent as required by their local IRB. Participants who are 18 years and older sign informed consent themselves. In addition to informed consent to participate in the trial, additional consent is obtained for the storage of biological specimens for subsequent analysis.

### Pharmacokinetics studies and determination of hydroxyurea dose

Once informed consent is obtained, all participants have a baseline PK visit. At this visit, participants take a single 20 mg/kg dose of liquid hydroxyurea prepared at each study site and PK microsamples are collected at 3 time points (15 min, 60 min, and 180 min) as described below. The liquid formulation is used for the PK studies to allow for a precise 20 mg/kg dose, but older participants are allowed to take capsules if they choose once hydroxyurea is prescribed. After the baseline visit, the participant does not start hydroxyurea until a study-determined starting dose is established and prescribed by their local provider, typically within 1–2 weeks of the PK visit. PK samples are shipped on dry ice to the central laboratory at CCHMC for measurement of hydroxyurea concentrations, determination of PK curve and area under the concentration-time curve (AUC), calculation of starting dose options for both arms, and randomization. Randomization results are blinded to the PI and staff involved in the recruitment and management of study participants until the study is complete.

Below, we describe several novel features of the study, including sparse PK sampling using microsampling devices, novel methods of measuring hydroxyurea concentrations, and determination of the optimal PK-guided dose for each individual participant.

#### Sparse PK sampling

Traditional PK sampling requires collection of 1–3 mL of venous blood at many time points over several drug half-lives. This collection frequency and relatively large-volume venous blood draws over 8–12 h is not practical in a clinical setting, particularly for infants and young children, notably the difficulties and intolerance of frequent venous blood draws in very young children and the inconvenience of having to remain in the hospital/clinic setting for a long period of time. Through the TREAT study, using historical data [23], we developed a population PK model and a sparse sampling strategy that accurately estimates hydroxyurea drug exposure using only three hydroxyurea concentrations measured at optimally designed times: 15 min, 60 min, and 3 h after hydroxyurea administration [31]. The number and timing of sample collection was selected based on known PK patters such that an accurate estimation of exposure could be made. Additionally, as we hope for this PK-guided dosing strategy to be ultimately clinically feasible, we found that the collection of 3 samples over 3 h was acceptable to both families and feasible within a clinical setting. The TREAT cohort demonstrated the feasibility and safety of this sampling strategy in young children and older adolescent/young adults at a single center [29]. The PK sampling strategy was an important feature of TREAT that resulted in high rates of enrollment with > 90% of children with SCA who initiated hydroxyurea during the study period agreeing to participate.

#### Microsampling and measurement of hydroxyurea concentrations

**Fig. 3:**
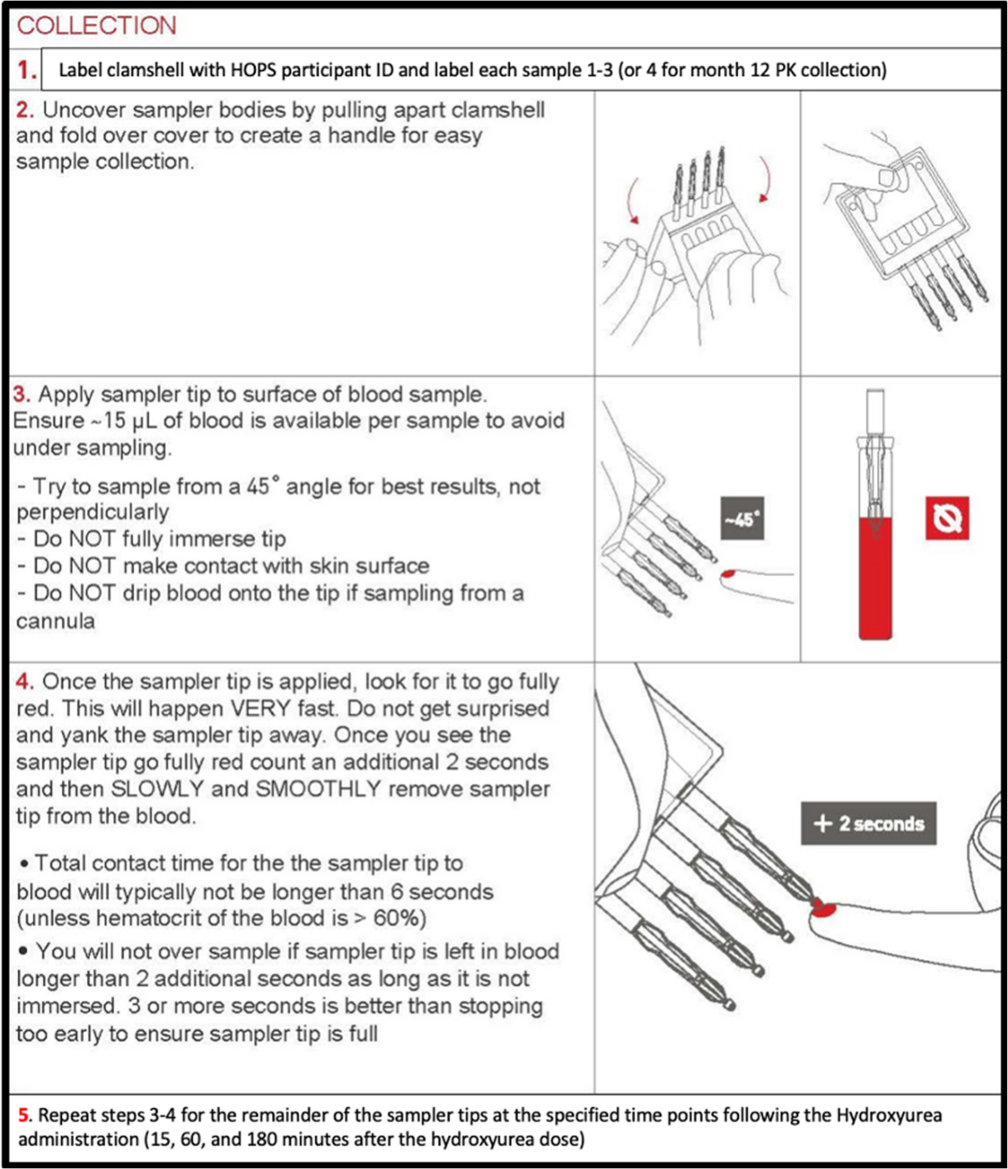
HOPS pharmacokinetics microsampling procedures. This figure, provided within the study Manual of Operations, details the sample collection process for pharmacokinetics samples collected by finger or heel stick using novel microsampling devices

There is no widely established method or commercially available technique for measuring hydroxyurea concentrations in biological samples, but several new and accurate techniques have been developed [32]. Our novel HPLC-based assay, requiring 0.5–1.0 mL of blood per time point, was the primary assay used in the TREAT study [29], and was a significant improvement from the previously used colorimetric assay, which required 1–2 mL per time point [33, 34]. We have since miniaturized the hydroxyurea assay further through the development of a highly sensitive and accurate tandem mass spectrometry-based assay (LC-MS/MS) for the quantitative measurement of hydroxyurea, requiring even smaller volumes of blood [35]. Blood collection occurs using novel Volumetric Absorption Microsampling (VAMS) devices (Neoteryx, LLC, Torrance, CA), which store exactly 10 μL and samples can be collected by finger stick or heel stick, which is much preferred compared to venous sampling, for young children. Figure 3 illustrates the microsampling collection process using these VAMS devices.

**Fig. 4:**
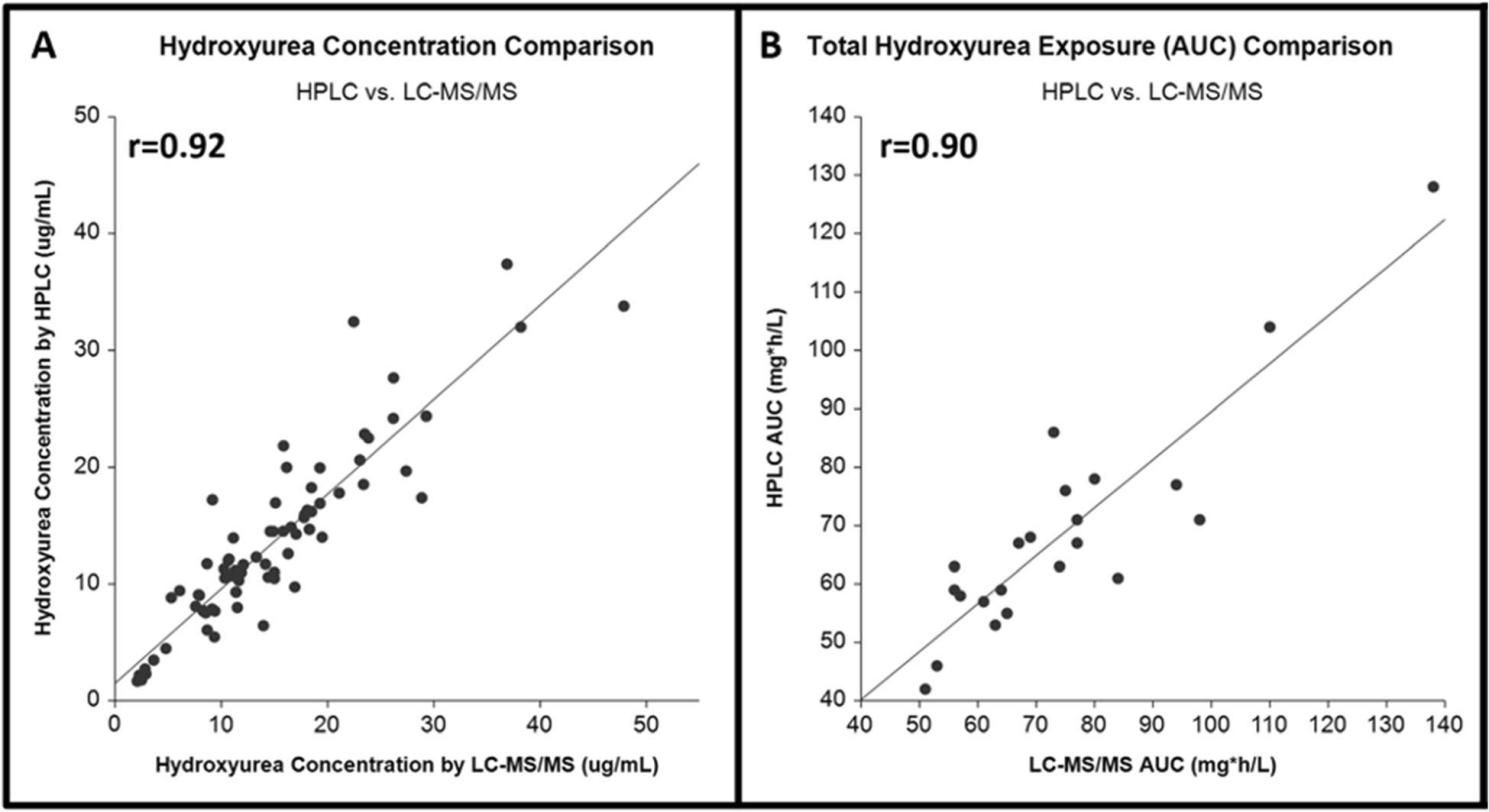
Comparison of methods to measure hydroxyurea concentrations. The novel LC-MS/MS method of hydroxyurea measurement was validated in comparison to the more standard HPLC technique. **a** The excellent correlation (*r* = 0.92) with individual hydroxyurea concentrations. **b** The correlation is similarly strong (*r* = 0.90) when each patient’s samples are combined using both measure to calculate hydroxyurea area under the concentration-time curve (AUC)

To validate this novel PK microsampling method, we compared hydroxyurea concentrations and AUC measurements using the established HPLC assay to the new LC-MS/MS assay. Among 80 samples from 23 TREAT participants, both methods gave similar hydroxyurea concentrations and AUC measurements (Fig. 4a, b, *r* > 0.90 for both comparisons). Importantly, this strong correlation in individual hydroxyurea concentrations also resulted in very similar recommended doses to target the desired AUC. PK-guided doses were calculated using data from both methods and demonstrated similar doses with a mean difference of − 1.7 ± 2.6 mg/kg in comparing the two methods. As hydroxyurea concentrations (and thus calculated AUC) tended to be slightly higher as measured by LC-MS/MS than HPLC, there were no recommended doses calculated using LC-MS/MS values that were greater than 3 mg/kg from the HPLC calculated dose. These data and the ease of collecting low volume samples using this technique provided confidence to use this technique as the primary method of hydroxyurea measurement for the HOPS trial.

For HOPS, PK samples are collected in duplicate using the described sparse sampling strategy (samples collected at 15 min, 60 min, and 180 min following the hydroxyurea dose) and the VAMS devices as shown in Fig. 3.

### Determination of PK-guided dose

As was done in the TREAT study, hydroxyurea concentrations are incorporated into the previously described population PK-model using MW/Pharm (Mediware, Prague, Czech Republic) [36]. To determine the PK-guided dose, each participant’s absorption profile is used to determine the dose that would achieve target AUC of 115 mg*h/L. This AUC target was determined through the analysis of PK studies performed in a cohort of children with SCA from the Hydroxyurea Study of Long-term Effects (HUSTLE, NCT00305175) after they reached a clinically-determined maximum tolerated dose [23, 31]. The optimal PK-guided dose is calculated and recorded for all participants, but only those participants randomized to the Alternative Arm would initiate hydroxyurea at this dose. At the conclusion of the study, the PK-guided dose calculated for the Standard Arm will be compared to the dose achieved through the dose escalation process. The PK-model allows for determination of a specific dose (in mg) that approximates an AUC of 115 mg*h/L, but at times, this dose is not convenient using common dosage forms (100 mg/mL liquid or 500 mg capsules). For participants who choose to take liquid hydroxyurea, the recommended starting doses (for both arms) are rounded to the nearest 20 mg (0.2 mL). For older participants who choose to take hydroxyurea capsules, a daily dose is selected that best approximates the recommended dose. This at times requires different doses on different days. For example, if a dose of 750 mg is recommended, the participant would alternate taking one (500 mg) and two (1000 mg) capsules each day for an average daily dose of 750 mg. Hydroxyurea will only be started if there are no baseline cytopenias as defined in the toxicity criteria in Table 1.

**Table 1:** Hydroxyurea dose adjustment and toxicity criteria

	Escalation criteria^**1**^	Criteria to adjust dose for weight gain^**2**^	Toxicity criteria^**3**^
**Neutrophils**	> 3.0 × 10^9^/L	> 1.5 × 10^9^/L	< 0.75 × 10^9^/L
**Platelets**	> 100 × 10^9^/L	> 120 × 10^9^/L	< 80 × 10^9^/L
**Reticulocytes and hemoglobin**	ARC> 50 × 10^9^/L if Hb > 7 g/dL	ARC> 100 × 10^9^/L if Hb < 8 g/dL ARC> 75 if Hb > 8 g/dL	ARC < 50 × 10^9^/L if Hb < 7 g/dL

^1^All laboratory criteria must be met to increase the dose

^2^All laboratory criteria must be met to adjust the dose. Dose adjustment is recommended when the current dose is ≤ 2.5 mg/kg from the starting dose

^3^Toxicity is defined and dose is held if any single one of these laboratory criteria occurs. If toxicity recurs or persists beyond 1 week, dose is decreased

### Randomization and blinding

Participants who complete their baseline PK visit are randomized in a 1:1 ratio to receive either hydroxyurea using a starting dose of 20 mg/kg/day (Standard Arm) or an individualized, PK-guided dose (Alternative Arm). All participants have both a standard (20 mg/kg) and PK-guided starting dose calculated and entered into a locked Research Electronic Data Capture (REDCap) form visible only to select members of the Data Coordinating Center, after which randomization occurs. The random allocation sequence is generated using the REDCap randomization module. The randomization procedure is stratified by age (age ≤ 2 and age > 2 years). This stratification for age is performed to increase the likelihood of having age balance in each treatment arm due to the fact that the primary endpoint (%HbF) is typically higher in children less than 2 years than in older children [6]. In the current era, guided by the 2014 NHLBI guidelines, most children with SCA are at least offered hydroxyurea and many begin taking hydroxyurea at a young age. We thus anticipate that the enrollment age for HOPS will be young, though likely not quite as young as the TREAT cohort given that there simply are not many older patients who have not yet been offered or started on hydroxyurea. The stratification for age is included to ensure each arm is balanced in terms of older and younger participants. Randomization is performed using a truncated binomial rule with permuted blocks, each 4 in size, within each stratum. The probability of assigning either treatment within each permuted block will be ½, until one of the two treatments has been assigned twice; all subsequent patients within the block receive the remaining treatment. This ensures that within each block as well as at the end of the study the treatment assignment is balanced. The Data Coordinating Center manager and REDCap data specialist, who are not involved in participant screening, enrollment, or assessment, are the only people with knowledge of the study arm. At this time, the local study team is informed that randomization has occurred and starting dose is available. The starting dose is provided as an absolute (mg) dose and entered into the REDCap study database.

The study is designed with the intent of a double-blind design, but is not formally labeled a “double-blind” trial due to the fact that hydroxyurea is used as an open-label study medication and the study team will know if the mg/kg dose is notably different than 20 mg/kg. Despite this possibility, the study arm is not explicitly provided to the provider or the family, and the same procedures are used for dose escalation or reduction throughout the remainder of the study. Additionally, because there are some patients on the Alternative (PK-guided) Arm who may have a dose that is very close to or the same as the Standard Arm dose of 20 mg/kg dose, it is not always possible to know the study arm assignment. Accordingly, we do not anticipate that lack of formal blinding will create bias in the treatment or outcomes of enrolled participants.

### Study-directed hydroxyurea dosing

**Fig. 5:**
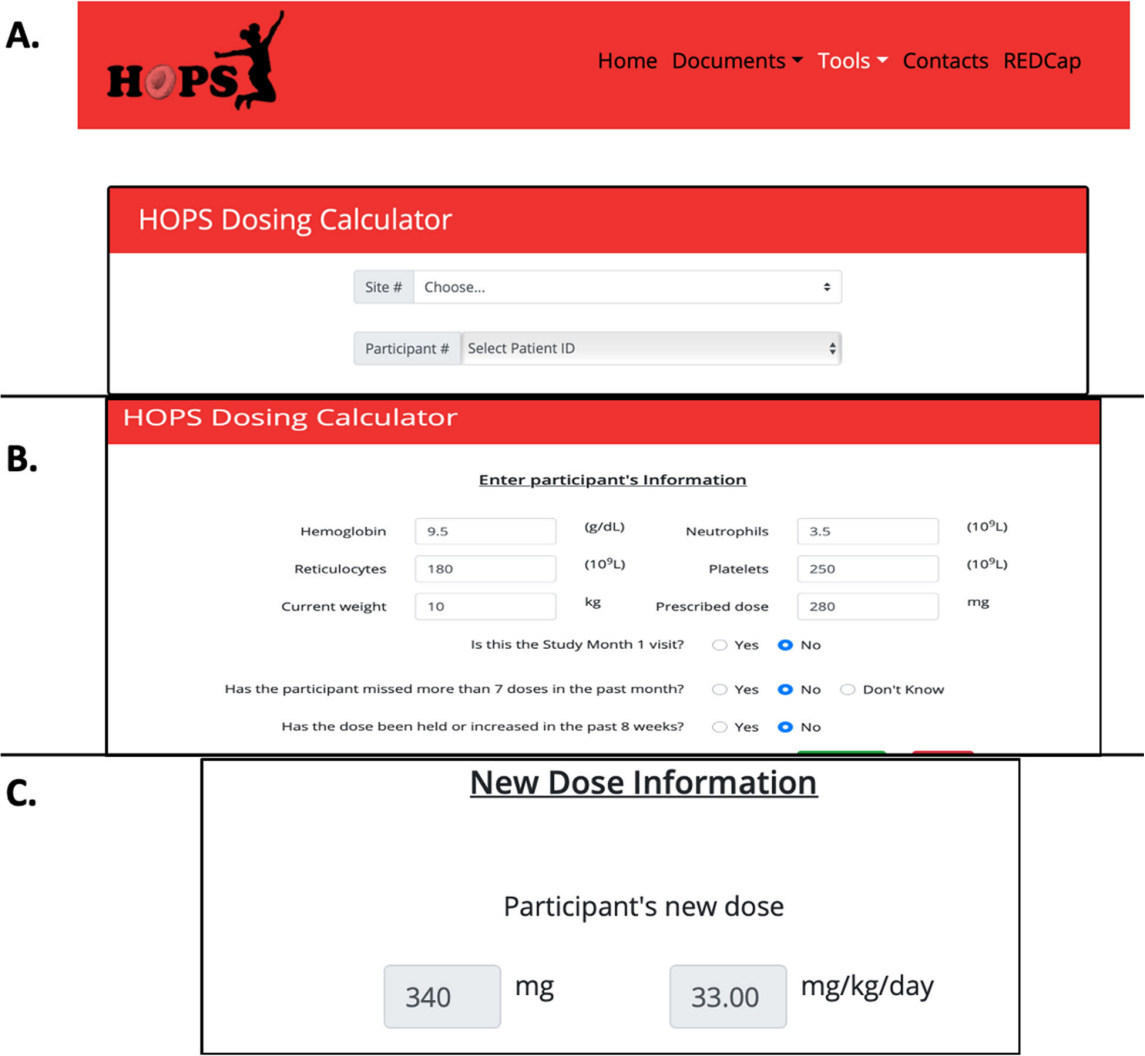
HOPS study dosing calculator. The HOPS study website includes a dosing calculator to allow for easy dose adjustments. Study personnel enter the Site # and Participant ID (**a**); current laboratory results, weight, and dosing information (**b**); and the calculator provides the new recommended dose (**c**)

The primary objective of HOPS is to compare PK-guided dosing to traditional, weight-based dosing. After selection of the starting dose, all participants, regardless of starting dose, are monitored and have dosage adjusted in the same way. The dose may be adjusted every 8 weeks based on laboratory values to target moderate myelosuppression. The maximum daily dose of hydroxyurea on the HOPS protocol will not exceed 35 mg/kg/day. The dose adjustment and toxicity criteria (Table 1) were decided upon through a consensus of study investigators and are less conservative than are used by most centers or previously published settings, tolerating lower absolute neutrophil, absolute reticulocyte, and platelet counts to optimize clinical benefits while still maintaining patient safety. This consensus decision was based on clinical experience that severe myelosuppression, even with the higher doses used in the TREAT trial, are uncommon with hydroxyurea therapy. With each study visit, participants’ prescribed dose and laboratory values are reviewed by their clinical provider who determines one of four options: (1) continue to prescribe hydroxyurea at the same dose, (2) escalate the prescribed dose, (3) adjust the prescribed dose to account for weight gain, or (4) temporarily hold and/or decrease the prescribed dose. Table 1 summarizes the dose adjustment and toxicity criteria. The study also created a HOPS Dosing Calculator, available on the study website to assist prescribers and to reduce the potential for variation in dosing regimens across study sites (Fig. 5). While the calculator is designed to guide dosing decisions, dosing decisions can rely upon clinical discretion at any time throughout the study. Medication adherence is encouraged and patients/caregivers self-report their adherence in person at study visits, by telephone, or if the family agrees, through an electronic REDCap survey sent automatically on a monthly basis by text message or e-mail. We recognize that suboptimal adherence is the primary barrier to effective hydroxyurea therapy, but we aim for this study to mimic “real-world” circumstances and purposefully did not include excessive efforts to address medication adherence.

### Outcome measures

The primary endpoint is the mean %HbF at 6 months, and participants who are randomized to either the Standard or Alternative Arm are included in the primary endpoint analysis according to the intent to treat principle. We hypothesize that the %HbF will be at least 5 percentage points higher (e.g., HbF of 25% compared to HbF of 30%) in children who initiate hydroxyurea at the PK-guided dose compared to those who start at a standard, 20 mg/kg dose. While there is no true perfect biomarker that predicts the morbidity and mortality of SCA, %HbF was chosen as the primary endpoint as it is the most well-established protective factor that is able to prevent polymerization of HbS and the subsequent complications of SCA. While traditional therapy often achieves modest levels of HbF, we aim to maximize the HbF response beyond the 30% level that has been postulated to be necessary to truly prevent HbS polymerization and RBC sickling. Although safety is not a primary study endpoint, we will carefully collect and analyze the frequency and severity of both laboratory and clinical adverse events, particularly cytopenias that may be the result of hydroxyurea dosing. We will also investigate the clinical, laboratory, and molecular determinants of the maximum hydroxyurea-induced HbF responses. Parameters such as age, sex, baseline (pre-treatment) %HbF, ANC, ARC, and the number of alpha-globin genes present will be analyzed, along with the optimal hydroxyurea dose, and selected PK (AUC, Cmax, t_1/2_) and PD (%HbF, Hb, MCV, ARC, ANC) variables. We also will analyze the epigenomic signature and gene expression patterns of study participants receiving hydroxyurea therapy after they have achieved the optimal dose, with the goal of elucidating the underlying mechanisms that determine optimal hydroxyurea dose and ultimate HbF response.

### Sample size and statistical analysis plan

A total of 116 patients will be enrolled (58 per arm). The difference in the mean %HbF at 6 months will be compared between treatments using a one-sided Welch’s two sample *t* test. Welch’s *t* test will be used due to evidence in the preliminary data that the standard deviations in the two treatment arms may different. A one-sided test is used due to the hypothesis, based on preliminary data, that the PK-guided dosing will have a higher HbF% than the standard, weight-based dosing arm. Significance (and superiority for the primary endpoint) will be assessed at the 0.05 level of significance. The power calculation is based on two separate concluded studies. For data representative of the Standard Arm, the SWITCH trial yielded a mean %HbF and SD of 29.1% and 6.7% post treatment, respectively [37]. For data representative of the Alternative Arm, the TREAT trial yielded a mean %HbF and SD of 34.7% and 9.9% post treatment, respectively [29]. For the purpose of power calculation, we used a conservative estimate of 5% for the mean %HbF difference and 7% and 10% for the SD for the Standard and Alternative Arm, respectively. Based on the preliminary data, a sample size of 104 (52 per arm) evaluable patients will have an approximate power of 0.90 and will control the one-sided error rate. Anticipating a 10% drop out rate, we will randomize 116 (58 per arm) patients to one of the two treatment arms. For the determination of predictors of %HbF response, multivariate linear-regression models will be used to identify independent predictors of ‘%HbF response. A *p* value of less than 0.05 will be considered statistically significant.

### Data collection methods and data management

CCHMC served as the Data Coordinating Center and the Data Management Team. Data is collected using standardized paper case report forms (CRFs), which is subsequently entered into a secured REDCap database hosted at Cincinnati Children’s Hospital Medical Center. REDCap is a secure, web-based software platform designed to support data capture for research studies, providing (1) an intuitive interface for validated data capture, (2) audit trails for tracking data manipulation and export procedures, (3) automated export procedures for seamless data downloads to common statistical packages, and (4) procedures for data integration and interoperability with external sources [38, 39]. The REDCap data capture system or equivalent electronic data capture system provides a platform for study-wide oversight of safety monitoring activities with automated notifications within the protocol context. Notifications are based on key study functions including adverse events serious adverse events tracking and CRF completion. These notifications and reporting tools are used to ensure timely communication between the study sites, protocol management, and coordinating center staff and that reporting requirements are met in all instances. All study data and biospecimens for the proposed studies will be collected directly from the study participants themselves, through a guardian for younger children in the case of questionnaires, or through the medical record for clinically obtained exams. Upon informed consent, participants are issued a study ID number and a numerical study identifier to be used throughout the remainder of the study to ensure confidentiality.

### Data monitoring

Regulatory and compliance study monitoring of HOPS clinical trials is a continuous, ongoing review of the conduct of this trial to ensure that it is conducted, documented, and reported in accordance with the Institutional Review Board (IRB)-approved protocol, the International Conference of Harmonisation (ICH) Good Clinical Practice Guidelines (GCPs), institutional policies, and applicable regulatory requirements.

Study monitoring includes monitoring for central elements defined as:

- Data related to primary study endpoints;
- Appropriateness of consent documentation;
- Protocol eligibility;
- Protocol compliance;
- Timeliness of data entry, including the reporting of adverse events (AE/AR) and serious adverse events (SAE/SSAR) as events are reported;
- Documentation of response assessment measures;
- Essential regulatory documentation; and
- Site investigator supervision of overall conduct of the study.

Regulatory and compliance monitoring includes a combination of on-site visits (scheduled to occur at least annually or more frequently based on enrollment, the degree of risk or severity of monitoring findings, and other study management issues) along with off-site ongoing (remote) efforts. The study has a specific Data Safety Monitoring Plan that includes a designated Medical Monitor unaffiliated with the study who regularly reviews safety data and all adverse and serious adverse events.

### Ethical considerations

Each clinical site uses locally approved informed consent documents, based upon the HOPS Model Consent Form (Additional file 1). Once a potentially eligible patient has been identified and approached, a consent conference takes place with the patient and family to discuss the study and explain the study procedures. The protocol is carefully described including the purpose of the study, risks and benefits of study participation, the treatment and assessments, and the patients’ rights and responsibilities if they enroll in the study, including the ability to withdraw from the study at any time. At a minimum, one parent or the legally authorized representative(s) is included in the conference. When the consent is signed, a consent process note must be written in the medical record or the research chart. The original consent document is filed in the participant’s research record and a signed copy of the consent document should be provided to the participant and/or LAR or as directed by the local ethical review board. The Informed Consent CRF is completed in REDCap at this time as well. In addition to the study consent, there is an optional consent to store blood for future research purposes. The willingness or unwillingness to sign this optional consent does not affect the ability of the child to receive the full breadth of study treatment and procedures.

### Study discontinuation

The following list provides the scenarios in which a HOPS participant may be removed from the protocol.

1. Initiation of chronic transfusions at any time after enrollment on the study
2. Parental or family decision to withdraw from the study
3. Participant decides to no longer participate in the study
4. Investigators may discontinue any participant at their discretion, if in their professional opinion, the participant’s health, safety, and/or well-being is threatened by continued participation in the study.
5. Participant decides not to initiate or to discontinue hydroxyurea treatment permanently
6. Participants who are not able to complete necessary study procedures. Specifically, if a participant has a 3-month (90 days) period without a complete blood count during the first 6 months of treatment, the participant may be removed from the study.
7. Death

If more than 10% of participants in either arm are removed from the study prior to the completion of 6 months of therapy as designed by the sample size estimates, we will recruit new participants to reach the required sample size of 104 participants.

## Discussion

Despite decades of evidence demonstrating the benefits of hydroxyurea as a disease-modifying treatment for SCA and national guidelines with strong recommendations to offer hydroxyurea starting at an early age, hydroxyurea remains underutilized [18, 20]. The underutilization is likely due in part to the perceived lack of clinical benefits by both providers and patients. Furthermore, suboptimal dosing of hydroxyurea may lead to medication non-adherence or perceived poor adherence by the provider and subsequently cause provider-patient mistrust. Despite the claims of sub-populations of patients with SCA who do not respond to hydroxyurea therapy, we feel strongly that the primary reason for suboptimal response may be inadequate hydroxyurea exposure (dose). Recent data from TREAT has demonstrated that early initiation of hydroxyurea using individualized, PK-guided dosing to maximize benefits and minimize toxicity has the potential to be close to a curative therapy with high levels and pancellular expression of HbF within red blood cells and not only the reduction in but elimination of clinical complications in most adherent patients. The HOPS trial aims to validate the encouraging results from the single center TREAT cohort in a multi-center, randomized, and partially blinded prospective clinical trial to determine if this approach is feasible, effective, and generalizable.

Although the study hypothesis is that PK-guided dosing will have improved laboratory response compared to weight-based initial dosing, the implementation of a personalized medicine approach for the pediatric sickle cell population and any study result will be important to understand and optimize treatment for children with SCA. If the PK-guided dosing arm demonstrates superior laboratory benefits, we will work toward making PK-guided dosing more widely available. The multi-center design of the study informs the feasibility of collecting and processing of micro-PK samples in a variety of clinical settings with centralized laboratory analysis. We will have a very clear and extensively tested standard operating procedure on the collection, processing, and shipment of PK samples that could easily be replicated in order to make PK-guided dosing more clinically available. It is important to note that although the “Standard” Arm for HOPS starts at a weight-based dose of 20 mg/kg/day, the dose escalation strategy is more aggressive than dosing guidelines recommended by the 2014 NHLBI guidelines and those used by most pediatric providers. Thus, the study results will provide important data to inform hydroxyurea dosing with or without the availability of PK-guided dosing.

The study has several limitations. The primary limitation is that the focus of this study is on children (mostly very young children) with SCA, who do not yet have chronic organ damage that is significant enough to affect hydroxyurea dosing or response. There are thus no specific inclusion or exclusion criteria related to renal sufficiency for the HOPS trial. In pediatrics, it is rare for hydroxyurea dose or response to be significantly affected by renal function. In contrast, adult patients with SCA, especially if they have had decades of untreated SCA, have significant organ damage, specifically to their kidneys and bone marrow. This results in the inability to tolerate even modest doses of hydroxyurea that subsequently results in suboptimal clinical response. Renal function will be measured in the HOPS cohort and secondary analyses will be performed to determine if renal function has an impact on hydroxyurea dosing or response, but this is not a primary objective of the current study. Thus, the study results may apply most specifically to young children with SCA. The HOPS trial will importantly demonstrate the feasibility of PK-guided dosing using a centralized laboratory, but we are simultaneously planning an additional hydroxyurea PK/PD study for adults with the goal of developing an individualized dosing model that takes renal function and bone marrow reserve into account. Another important limitation of the study is that study participants may have increased medication adherence during the relatively short study due to being enrolled in a clinical trial and due to prompting by the regular study-based reminders about hydroxyurea adherence. Further investigation of the long-term sustainability of this dosing strategy will be importance, but medication adherence efforts are not a primary focus of the current study.

Finally, the HOPS trial is important as it aims to set a new standard in what is becoming a new generation of hydroxyurea therapy. When hydroxyurea was first introduced decades ago, there was tremendous skepticism regarding its safety and benefits. For these reasons, there were stringent clinical criteria that had to be met when starting hydroxyurea and dosing was very conservative with absolute neutrophil count goals of > 4.0 × 10^9^/L (compared to ~ 1.5 × 10^9^/L in HOPS). The BABY HUG study was a critical, paradigm-shifting study that demonstrated the benefits in otherwise “asymptomatic” infants and young children with SCA compared to placebo. Since the publication of the BABY HUG results and the subsequent NHLBI guidelines to use hydroxyurea beginning within the first year of life [18, 40], hydroxyurea is slowly being considered the standard of care. Rather than describing hydroxyurea as a medication that will be used only if complications develop, providers discuss hydroxyurea as a critical medication as a medication that can be started in the first year of life to protect against both short- and long-term complications. HOPS has the opportunity to provide important evidence to guide the dosing of hydroxyurea for these young children. We hypothesize that both arms of the HOPS trial will have more pronounced laboratory benefits when compared to the BABY HUG cohort, who received hydroxyurea at a fixed 20 mg/kg/day dose. Despite the increase in the number of new medications in the sickle cell space, hydroxyurea remains the primary disease-modifying therapy and therefore, it is essential to determine the optimal hydroxyurea dosage strategy and age to initiate hydroxyurea as these could result in a new generation of children with few, if any, SCA complications.

## Trial status

The study is currently approved and actively enrolling participants at 10 of 11 study sites. The current approved protocol is version 2.0 (version date October 23, 2019). The first participant enrolled on June 13, 2019. The COVID-19 pandemic has slowed recruitment, but we anticipate study recruitment will be completed by mid-2021. The careful selection and education process for each site, coupled with enthusiastic and proactive local investigators has allowed for the study to succeed in this early phase. The preliminary results from TREAT and the novel dosing strategy have allowed study investigators to have informed conversations with families regarding the low risk and possible benefits of study enrollment. Whereas there has been concern with slow enrollment of sickle cell patients in clinical trials, this has not been our experience with TREAT or in the early phases of HOPS. The HOPS study team has regular virtual meetings to review study procedures and to share challenges and experience across sites.

## Supplementary Information

**Additional file 1.**

## Data Availability

The results from this clinical trial have the potential to change dosing of hydroxyurea for sickle cell patients. The dataset will be shared among study investigators for analysis and will be disseminated through publications and oral presentations. All plans for dissemination of study results will be discussed with the study investigators prior to implementation.

## Abbreviations

AUC: Area under the concentration-time curve
CRF: Case report form
HbF: Fetal hemoglobin
HOPS: Hydroxyurea Optimization through Precision Study
NHLBI: National Heart, Lung, and Blood Institute
PD: Pharmacodynamics
PK: Pharmacokinetics
REDCap: Research Electronic Data Capture
SCD: Sickle cell disease
SCA: Sickle cell anemia
TREAT: Therapeutic Response Evaluation and Adherence Trial
VAMS: Volumetric Absorption Microsampling

## References

[1] Piel FB, Hay SI, Gupta S, Weatherall DJ, Williams TN (2013). Global burden of sickle cell anaemia in children under five, 2010-2050: modelling based on demographics, excess mortality, and interventions. PLoS Med.

[2] Piel FB, Patil AP, Howes RE (2013). Global epidemiology of sickle haemoglobin in neonates: a contemporary geostatistical model-based map and population estimates. Lancet.

[3] Hassell KL (2010). Population estimates of sickle cell disease in the U.S. Am J Prev Med.

[4] Gill FM, Sleeper LA, Weiner SJ (1995). Clinical events in the first decade in a cohort of infants with sickle cell disease. Cooperative Study of Sickle Cell Disease. Blood.

[5] Ohene-Frempong K, Weiner SJ, Sleeper LA (1998). Cerebrovascular accidents in sickle cell disease: rates and risk factors. Blood.

[6] Marcus SJ, Ware RE (1999). Physiologic decline in fetal hemoglobin parameters in infants with sickle cell disease: implications for pharmacological intervention. J Pediatr Hematol Oncol.

[7] Brousse V, Buffet P, Rees D (2014). The spleen and sickle cell disease: the sick(led) spleen. Br J Haematol.

[8] Quinn CT (2013). Sickle cell disease in childhood: from newborn screening through transition to adult medical care. Pediatr Clin N Am.

[9] Platt OS (2008). Hydroxyurea for the treatment of sickle cell anemia. N Engl J Med.

[10] Charache S, Terrin ML, Moore RD (1995). Effect of hydroxyurea on the frequency of painful crises in sickle cell anemia. Investigators of the Multicenter Study of Hydroxyurea in Sickle Cell Anemia. N Engl J Med.

[11] Strouse JJ, Heeney MM (2012). Hydroxyurea for the treatment of sickle cell disease: efficacy, barriers, toxicity, and management in children. Pediatr Blood Cancer.

[12] Lanzkron S, Strouse JJ, Wilson R (2008). Systematic review: Hydroxyurea for the treatment of adults with sickle cell disease. Ann Intern Med.

[13] Steinberg MH, McCarthy WF, Castro O (2010). The risks and benefits of long-term use of hydroxyurea in sickle cell anemia: a 17.5 year follow-up. Am J Hematol.

[14] Le PQ, Gulbis B, Dedeken L (2015). Survival among children and adults with sickle cell disease in Belgium: benefit from hydroxyurea treatment. Pediatr Blood Cancer.

[15] Voskaridou E, Christoulas D, Bilalis A (2010). The effect of prolonged administration of hydroxyurea on morbidity and mortality in adult patients with sickle cell syndromes: results of a 17-year, single-center trial (LaSHS). Blood.

[16] Lobo CL, Pinto JF, Nascimento EM, Moura PG, Cardoso GP, Hankins JS (2013). The effect of hydroxcarbamide therapy on survival of children with sickle cell disease. Br J Haematol.

[17] McGann PT, Ware RE (2011). Hydroxyurea for sickle cell anemia: what have we learned and what questions still remain?. Curr Opin Hematol.

[18] Yawn BP, Buchanan GR, Afenyi-Annan AN (2014). Management of sickle cell disease: summary of the 2014 evidence-based report by expert panel members. JAMA.

[19] Schuchard SB, Lissick JR, Nickel A (2019). Hydroxyurea use in young infants with sickle cell disease. Pediatr Blood Cancer.

[20] Brousseau DC, Richardson T, Hall M (2019). Hydroxyurea use for sickle cell disease among Medicaid-enrolled children. Pediatrics.

[21] Su ZT, Segal JB, Lanzkron S, Ogunsile FJ (2019). National trends in hydroxyurea and opioid prescribing for sickle cell disease by office-based physicians in the United States, 1997-2017. Pharmacoepidemiol Drug Saf.

[22] Ware RE (2015). Optimizing hydroxyurea therapy for sickle cell anemia. Hematol Am Soc Hematol Educ Program.

[23] Ware RE, Despotovic JM, Mortier NA (2011). Pharmacokinetics, pharmacodynamics, and pharmacogenetics of hydroxyurea treatment for children with sickle cell anemia. Blood.

[24] Paule I, Sassi H, Habibi A (2011). Population pharmacokinetics and pharmacodynamics of hydroxyurea in sickle cell anemia patients, a basis for optimizing the dosing regimen. Orphanet J Rare Dis.

[25] Alvarez O, Miller ST, Wang WC (2012). Effect of hydroxyurea treatment on renal function parameters: results from the multi-center placebo-controlled BABY HUG clinical trial for infants with sickle cell anemia. Pediatr Blood Cancer.

[26] Ware RE, Rees RC, Sarnaik SA (2010). Renal function in infants with sickle cell anemia: baseline data from the BABY HUG trial. J Pediatr.

[27] Steinberg MH, Chui DH, Dover GJ, Sebastiani P, Alsultan A (2014). Fetal hemoglobin in sickle cell anemia: a glass half full?. Blood.

[28] Buchanan GR (2014). “Packaging” of fetal hemoglobin in sickle cell anemia. Blood.

[29] McGann PT, Niss O, Dong M (2019). Robust clinical and laboratory response to hydroxyurea using pharmacokinetically guided dosing for young children with sickle cell anemia. Am J Hematol.

[30] Shook LM, Farrell CB, Kalinyak KA (2016). Translating sickle cell guidelines into practice for primary care providers with Project ECHO. Med Educ Online.

[31] Dong M, McGann PT, Mizuno T, Ware RE, Vinks AA (2016). Development of a pharmacokinetic-guided dose individualization strategy for hydroxyurea treatment in children with sickle cell anaemia. Br J Clin Pharmacol.

[32] Marahatta A, Ware RE (2017). Hydroxyurea: analytical techniques and quantitative analysis. Blood Cells Mol Dis.

[33] Heeney MM, Whorton MR, Howard TA, Johnson CA, Ware RE (2004). Chemical and functional analysis of hydroxyurea oral solutions. J Pediatr Hematol Oncol.

[34] Fabricius E, Rajewsky F (1971). Determination of hydroxyurea in mammalian tissues and blood. Rev Eur Etud Clin Biol.

[35] Marahatta A, Megaraj V, McGann PT, Ware RE, Setchell KD (2016). Stable-isotope dilution HPLC-electrospray ionization tandem mass spectrometry method for quantifying hydroxyurea in dried blood samples. Clin Chem.

[36] Proost JH, Meijer DK (1992). MW/Pharm, an integrated software package for drug dosage regimen calculation and therapeutic drug monitoring. Comput Biol Med.

[37] Ware RE, Helms RW, Investigators SW (2012). Stroke with transfusions changing to hydroxyurea (SWiTCH). Blood.

[38] Harris PA, Taylor R, Minor BL (2019). The REDCap consortium: Building an international community of software platform partners. J Biomed Inform.

[39] Harris PA, Taylor R, Thielke R, Payne J, Gonzalez N, Conde JG (2009). Research electronic data capture (REDCap)--a metadata-driven methodology and workflow process for providing translational research informatics support. J Biomed Inform.

[40] Wang WC, Ware RE, Miller ST (2011). Hydroxycarbamide in very young children with sickle-cell anaemia: a multicentre, randomised, controlled trial (BABY HUG). Lancet.

