# Supplementary material for: Hydroxyurea Optimization through Precision Study (HOPS): study protocol for a randomized, multicenter trial in children with sickle cell anemia
Source: Trials. 2020 Nov 27;21:983. doi: 10.1186/s13063-020-04912-z (PMC7691962; doi:10.1186/s13063-020-04912-z)
Supplement: Supplementary file 1 — Additional file 1. [file 13063_2020_4912_MOESM1_ESM.pdf]

CINCINNATI CHILDREN'S HOSPITAL MEDICAL CENTER
CONSENT TO PARTICIPATE IN A RESEARCH STUDY

STUDY TITLE: Hydroxyurea Optimization through Precision Study (**HOPS**): A prospective, multi-center, randomized trial of personalized, pharmacokinetics (PK)-guided dosing of hydroxyurea versus standard weight-based dosing for children with sickle cell anemia

FUNDING ORGANIZATION: Cincinnati Children's Hospital Medical Center

Patrick T. McGann, MD, MS
Name of Principal Investigator

513-636-4200 (ask for the Hematologist on call)
Telephone Number

INTRODUCTION

This consent form is written for the parent/legal guardian (also known as the caregiver) of the child who agrees to take part in this study, a participant who is 18 years old, or for a participant who has reached 18 years of age while on study.

Throughout this document, "you" stands for the research participant whether they are an adult or a child. The signatures at the end will clarify whether the research participant consented themselves or whether a parent gave permission for participation.

If you are currently participating in this study and have recently turned 18 years old, the information in this form describes the study that you have already begun. Since you are now 18, you will be asked if you wish to continue.

WHY ARE WE DOING THIS RESEARCH?

In this research study we want to learn more about treatment of sickle cell anemia (SCA).

We are asking you and other people with SCA, who have chosen to start hydroxyurea, to participate in the research study because we want to compare the standard dosing of hydroxyurea to a new, individualized way of dosing. We are studying whether this new dosing strategy can result in even better results than standard dosing. Hydroxyurea is usually dosed based only on your weight. Our study will use a new way to select a starting dose that is based on how each patient absorbs hydroxyurea. We have had success with a smaller, initial research study at Cincinnati Children's Hospital that tested this dosing strategy on children with SCA.

It is estimated about 116 children and adults will participate in this study at Cincinnati Children's Hospital Medical Center (CCHMC) and other centers in the United States. Approximately 15 participants will take part in this study at CCHMC.

WHO IS IN CHARGE OF THE RESEARCH?

Dr. Patrick McGann is the researcher at Cincinnati Children's Hospital Medical Center (CCHMC) that is in charge of this study.

WHO SHOULD NOT BE IN THIS STUDY?
You should not be in this study if:

- You have not already decided to take hydroxyurea;
- If you have already been treated with hydroxyurea within the last 3 months;
- If you are not sure you will take hydroxyurea as prescribed;
- If you are unable to have your blood drawn every month and return to clinic as scheduled;
- Because we will be following you for one year after starting hydroxyurea, you should not be in the study if you are planning to move out of the area or transition to another hospital for care within the next year; or
- If you are participating in any other research studies that include investigational medications for your SCA.

Your study doctor will discuss with you any other concerns you may have about participating in this study.

WHAT WILL HAPPEN IN THE STUDY?
The research staff will explain each visit to you in detail. You will be able to ask questions to make sure that you understand what will happen.

After talking to your doctor and deciding that you want to take hydroxyurea for management of your SCA and agreeing to participate in this study, you will be asked to come to the hospital nine times over the next year for routine monitoring of your blood counts. This is not a significantly higher number of visits or blood draws than would be required for routine hydroxyurea monitoring even if you did not participate in this study.

Baseline Visit
You should plan to spend approximately four hours at CCHMC for this visit.

At this visit, as with any doctor’s visit, your doctor will perform a physical exam and will review your medical history.

We will ask you to take a single liquid dose of hydroxyurea at this visit. This dose is the usual starting dose of hydroxyurea, 20 mg/kg, based on your weight on this day.

After taking this dose, you will have three blood samples collected over 3 hours. The first blood draw will be 15 minutes after taking the dose of hydroxyurea, the second will be 60 minutes, and the third will be 3 hours from taking the dose of hydroxyurea. All three blood samples, can be drawn by pricking your finger or heel. One blood draw will be by vein and include other baseline lab studies. We will use these blood samples to measure the amount of hydroxyurea in your blood and to learn how your body absorbs and gets rid of the hydroxyurea after you take it.

In total, this first visit will require the collection of not more than 3 teaspoons of blood.

Randomization
Because we want to learn if this new dosing strategy is effective, the study will “randomize” each participant to receive the personalized dose or the standard dose. Being randomized means you will be put into a study group by chance, like flipping a coin. You will have an equal

chance of being in either study group, standard or alternative starting dose. Half of the participants in the study will receive the personalized starting dose and the other half of participants will receive the standard starting dose based upon their weight.

The dose of hydroxyurea you will receive will be based on the group to which you have been randomized. The dose will be changed as needed based on your response to the drug.

You will not have to come back to the hospital for randomization. The study sponsor will send your starting dose to your doctor usually within 2 weeks of your baseline visit. Your doctor will then contact you to let you know that your prescription is ready.

Monthly Lab Draws and Clinic Visits (Months 1-9)
You will see your doctor at least every 3 months throughout the study. Most visits for the study will happen the same time you are already scheduled with your doctor, but it is possible we may have you come in for a blood draw if you miss any routine clinic visits.

Beginning one month after receiving your prescribed dose of hydroxyurea, you will be asked to have less than a teaspoon of blood drawn once a month for the first six months and at month 9. Your study doctor may change or hold your dose of hydroxyurea based on the results of these blood draws. If your study doctor holds your dose of hydroxyurea, you will need to come back to the clinic to have your blood drawn again.

At month 6, some of the blood collected will be used for special research tests to see if you have a response to hydroxyurea.

In addition to blood work, during months 3, 6, and 9, your study doctor will review your medical history and perform a physical exam to see how you are feeling and to see if you have had any sickle cell problems.

Month 12 (End of Study Visit)
We will ask you to complete study related activities similarly to the baseline visit and you should plan to spend approximately four hours at CCHMC for this final study visit.

Your study doctor will complete a physical exam, a review of your medical history, and complete your final blood draw. We will repeat the blood testing of hydroxyurea like with the first visit. To do this, we will ask that you bring to clinic your prescribed dose of hydroxyurea. Before you take your prescribed dose of hydroxyurea, we will collect a small amount of blood to compare to the rest of your blood samples this day.

Then you will take your prescribed dose of hydroxyurea and have blood drawn after 15 minutes, 60 minutes, and 3 hours. These four blood draws will require a very small amount (a drop) of blood that can be drawn by pricking your finger or heel.

One blood draw will be by vein and include other lab studies. We will use these blood samples to measure the amount of hydroxyurea in your blood and to learn how your body absorbs and gets rid of the hydroxyurea after you take it.

In total, this last visit will require the collection of not more than 3 teaspoons of blood.

Adherence Survey
In addition to learning how your body and SCA work with hydroxyurea, we would like to know how you take your medicine at home. You will be asked to complete a small questionnaire.

You will be asked to complete this questionnaire once a month for the first six months after you start taking your prescribed dose of hydroxyurea at home and again at month 9 and 12. You will have the option to complete this questionnaire in clinic or if you prefer, this can be done by phone or email. Your answers to the questionnaire will be recorded in a secure electronic database and be shared with the study sponsor.

If at any time you do not want to answer these questions, you can let your study doctor or his research team know and you do not have to answer.

Additional Specimens
After doing the routine and required special research tests on your blood, as described above during the baseline and month 6 visit, we would like to store and save any leftover blood for future research studies, including studies on your genetic material (called DNA). These tests will help us to understand how different patients have different doses, different absorption, and different responses to hydroxyurea. We would store these samples indefinitely in the study's research laboratory. No results of any research studies done in the future on your blood will be available to you.

You may choose whether or not to allow our blood to be used for future research. No matter what you decide to do about the use of your samples, you can still take part in this study. Storing and sharing the blood samples is optional. Please initial in the spaces below next to your choice.

________ / ________<br>**Initials & Date**	**Yes**, I agree to allow leftover blood and genetic material (DNA) to be stored and shared for future research.
________ / ________<br>**Initials & Date**	**No**, I do not agree to allow leftover blood and genetic material (DNA) to be stored and shared for future research.

WHAT ARE THE GOOD THINGS THAT CAN HAPPEN FROM THIS RESEARCH?
Standard dosing of hydroxyurea will provide significant benefits to participants with SCA.

We cannot guarantee if you are randomized to the study arm using personalized dosing, it will result in improved benefits compared to standard dosing of hydroxyurea. However, when we finish the study, we hope that we will know more about hydroxyurea and SCA treatment.

WHAT ARE THE BAD THINGS THAT CAN HAPPEN FROM THIS RESEARCH?
Hydroxyurea
As part of your decision to begin hydroxyurea, you have already had a discussion with your doctor about the risks and benefits of hydroxyurea. There are minimal additional risks for you in this study. One of the important goals of this study is to accurately predict the best dose of hydroxyurea. We hope that this predicted dose will be accurate and will not cause any

problems, but there is a possibility that the prescribed starting dose may be slightly too high. If the dose is too high, some of your blood counts may decrease and your dose may need to be adjusted.

We will monitor your blood counts closely, especially in the first six months of treatment to be sure to adjust the dose if needed. If your blood counts do show that the dose is too high, we will ask that you stop taking hydroxyurea for one week until you can return to clinic where your blood counts will be repeated. If your blood counts remain low after 2 weeks, we will reduce your dose.

Although very uncommon, there is a slight risk that a decreased white blood count could increase the risk of infection. These risks are why we monitor blood counts so regularly for participants taking hydroxyurea. If the blood counts decrease too much and you have fever, your doctor will provide appropriate treatment.

Blood Sample Risks

When we take a blood sample, the needle may hurt or cause some bruising in the area where the blood was drawn. There is a very small chance that you may get an infection where the needle went in. We will give you medicine if infection occurs. Some people faint when their blood is drawn.

WHAT OTHER CHOICES ARE THERE?

Instead of being in this study, you can choose not to be in it. If you choose to not be in this study, your clinical care will not be changed. You can still receive hydroxyurea treatment clinically even if you choose not to participate in this research study.

HOW WILL INFORMATION ABOUT YOU BE KEPT PRIVATE?

Making sure that information about you remains private is important to us. To protect your privacy in this research study we will label your data with a code instead of your name. Only people involved in the study will have access to your personal information during this study.

A description of this clinical trial will be available on http://www.ClinicalTrials.gov, as required by U.S. Law. This website will not include information that can identify you. At most, the website will include a summary of the study results. You can search this website at any time.

The Food and Drug Administration (FDA) may choose to inspect your child’s records since your child is a participant in this research study.

WHAT IF WE LEARN NEW INFORMATION DURING THE RESEARCH?

The study doctor will tell you if they find out about new information from this or other studies that may affect your health, safety or willingness to stay in this study.

WILL IT COST YOU ANYTHING EXTRA TO BE IN THE RESEARCH STUDY?

The first dose of hydroxyurea taken at your first clinic visit will be provided free of cost to you as part of the research study.

Since you and your doctor have decided that you will take hydroxyurea to clinically manage your SCA, any hydroxyurea you take after your first clinic visit will be billed to your health insurance or Medicaid/Medicare as if you were not participating in this study.

Additionally, any blood work done as part of routine clinical care will be billed to insurance. CCHMC will cover the cost of any blood work done for research purposes. Routine clinical and laboratory follow-up performed by the provider for routine clinical purposes will not be covered by CCHMC. The blood work done for research only purposes are testing:

- Pharmacokinetics (how hydroxyurea is absorbed by your body);
- DNA and RNA serum (to identify any genetic changes after starting hydroxyurea);
- Advanced blood test using the Advia machine (an advanced CBC);
- Cystatin C (to know how well your kidneys are working); and
- F cell measurements (measurement of oxygen levels in your blood cells)

Should you have any questions regarding the cost of hydroxyurea and any other clinical care costs, you should contact your insurance provider.

WILL YOU BE PAID TO BE IN THIS RESEARCH STUDY?
You will be compensated for your time and effort while you are in this research study.

For your time, you will be paid $50 for the baseline and month 12 visit, $20 for months 3, 6, and 9, and $10 for the blood draw only visits at months 1, 2, 4, and 5.

You will receive payment for this study in the form of a reloadable debit card (Clincard). We will give you a handout that will explain how to use the card. Because you are being paid for your participation, CCHMC is required by the Internal Revenue Service (IRS) to collect and use your social security number (SSN) or taxpayer identification number (TIN) to track the amount of money that we pay you. You will need to complete a Federal W-9 form for this income tax reporting. This form requires your Social Security number. This form will be given to the CCHMC business office. It will not be kept as part of your study chart. If you move, you will need to complete another W-9 with an updated address.

WHAT HAPPENS IF YOU ARE INJURED FROM BEING IN THIS STUDY?
If you believe that you have been injured as a result of this research you should contact Dr. Patrick McGann, at the number on the first page of this consent form, as soon as possible to discuss the concerns. Treatment for injuries is available at CCHMC or your local hospital. If you go to the Emergency Room or to another hospital or doctor it is important that you tell them that you are in a research study about hydroxyurea. If possible, you should give them a copy of this consent form.

CCHMC follows a policy of making all decisions about compensation for the medical treatment of physical injuries that happened during or were caused by research on an individual basis.

WHO DO YOU CALL IF YOU HAVE QUESTIONS OR PROBLEMS?
For questions, concerns, or complaints about this research study you can contact the study person listed on page 1 of this document.

If you would like to talk to someone that is not part of the research staff or if you have general questions about your research study rights or questions, concerns, or complaints about the research, you can call the CCHMC Institutional Review Board at 513-636-8039.

AUTHORIZATION FOR USE/DISCLOSURE OF HEALTH INFORMATION FOR RESEARCH

To be in this research study you must also give your permission (or authorization) to use and disclose (or share) your "protected health information" (called PHI for short).

What protected health information will be used and shared during this study?

CCHMC will need to use and share your PHI as part of this study. This PHI will come from:

- Your CCHMC medical records
- Your research records

The types of information that will be used and shared from these records include:

- Laboratory test results, diagnosis, and medications
- Reports and notes from clinical and research observations
- Imaging (like CT scans, MRI scans, x-rays, etc.) studies and reports
- If applicable, information concerning HIV testing or the treatment of AIDS or AIDS-related conditions, drug or alcohol abuse, drug-related conditions, alcoholism, and/or psychiatric/psychological conditions (but not psychotherapy notes).

Who will share, receive and/or use your protected health information in this study?

- Staff at all the research study sites (including CCHMC)
- Personnel who provide services to you as part of this study
- Other individuals and organizations that need to use your PHI in connection with the research, including people at the sponsor and organizations that the sponsor may use to oversee or conduct the study.
- The members of the CCHMC Institutional Review Board and staff of the Office of Research Compliance and Regulatory Affairs.

How will you know that your PHI is not misused?

People that receive your PHI as part of the research are generally limited in how they can use your PHI. In addition, most people who receive your PHI are also required by federal privacy laws to protect your PHI. However, some people that may receive your PHI may not be required to protect it and may share the information with others without your permission, if permitted by the laws that apply to them.

Can you change your mind?

You may choose to withdraw your permission at any time. A withdrawal of your permission to use and share your PHI would also include a withdrawal from participation in the research study. If you wish to withdraw your permission to use and share PHI you need to notify the study doctor, listed on the first page of this document, in writing. Your request will be effective immediately and no new PHI about you will be used or shared. The only exceptions are (1) any use or sharing of PHI that has already occurred or was in process prior to you withdrawing your permission and (2) any use or sharing that is needed to maintain the integrity of the research.

Will this permission expire?

Your permission will expire at the end of the study. If the study involves the creation or maintenance of a research database repository, this authorization will not expire.

Will your other medical care be impacted?
By signing this document you are agree to participate in this research study and give permission to CCHMC to use and share your PHI for the purpose of this research study. If you refuse to sign this document you will not be able to participate in the study. However, your rights concerning treatment not related to this study, payment for services, enrollment in a health plan or eligibility of benefits will not be affected.

SIGNATURES
The research team has discussed this study with you and answered all of your questions. Like any research, the researchers cannot predict exactly what will happen. Once you have had enough time to consider whether you should participate in this research you will document your consent by signature below.

You will receive a copy of this signed document for your records.

____
Printed Name of Research Participant

____________________________________ ____________
Signature of Research Participant Indicating Consent or Assent — Date

____________________________________ ____________
Signature of Parent or Legally Authorized Representative* — Date

____
* If signed by a legally authorized representative, a description of such representative's authority must be provided

____________________________________ ____________
Signature of Individual Obtaining Consent — Date
